# Effects of the Oral Angiotensin II Type 2 Receptor Agonist C21 in Sugen-Hypoxia Induced Pulmonary Hypertension in Rats

**DOI:** 10.3390/ijms24087478

**Published:** 2023-04-19

**Authors:** Göran Tornling, Rohit Batta, Dan Salvail, Johan Raud, Christopher P. Denton

**Affiliations:** 1 Respiratory Medicine Division, Department of Medicine Solna, Karolinska Institutet, 17177 Stockholm, Sweden; 2 Vicore Pharma AB, 11127 Stockholm, Sweden; 3 IPS Therapeutique Inc., Sherbrooke, QC J1L 2T9, Canada; 4 Institute of Environmental Medicine, Karolinska Institutet, 17177 Stockholm, Sweden; 5 Centre for Rheumatology, Royal Free Hospital, University College Medical School, London NW3 2PS, UK

**Keywords:** pulmonary hypertension, vascular remodeling, pulmonary fibrosis, renin-angiotensin system, angiotensin type 2 receptor agonist, C21, buloxibutid

## Abstract

Substantial evidence supports the involvement of the renin-angiotensin system in pulmonary hypertension (PH), and the angiotensin II type 2 receptor (AT_2_R) is known to exert tissue protective actions. The effect of the selective AT_2_R agonist C21 (also known as Compound **21** or buloxibutid) was evaluated in the rat Sugen-hypoxia PH model. After a single injection of Sugen 5416 and hypoxia for 21 days, C21 (2 or 20 mg/kg) or vehicle was administered perorally twice daily from Day 21 to Day 55. On Day 56, hemodynamic assessments were performed, and lung and heart tissue were prepared for quantification of cardiac and vascular remodeling and fibrosis. Treatment with C21 20 mg/kg improved cardiac output and stroke volume and decreased right ventricular hypertrophy (all *p* < 0.05). Treatment with C21 2 mg/kg significantly decreased vessel wall and muscular layer thickness and increased the luminal opening in vessels >100 μm (all *p* < 0.05). There were no significant differences between the two C21 doses on any parameter, and post hoc analyses comparing the merged C21 groups with the vehicle group showed that C21 treatment reduced vascular remodeling (reduced endothelial proliferation and thickening of the vascular wall) in vessels of all sizes; moreover, the diastolic pulmonary artery pressure and right ventricular pressure were reduced along with reduction of right ventricular hypertrophy. Sugen 5416 and hypoxia increased pulmonary collagen deposition, which was counteracted by C21 20 mg/kg. In conclusion, the effects of C21 on vascular remodeling, hemodynamic alterations, and fibrosis suggest that AT_2_R agonists may have a role in Group 1 and 3 PH treatment.

## 1. Introduction

Pulmonary hypertension carries a poor prognosis and remains a potentially fatal disease. It has been estimated that the prevalence of pulmonary hypertension is about 1% in the global population and up to 10% in individuals aged more than 65 years [1]. The current definition following the 6th World Symposium on Pulmonary Hypertension is a mean pulmonary arterial pressure (mPAP) >20 mmHg associated with an abnormal pulmonary vascular resistance (PVR) of ≥3 Wood units [2]. Based on etiology and pathophysiology, pulmonary hypertension (PH) is classified into five groups: (1) Pulmonary arterial hypertension (PAH), (2) PH due to left heart disease, (3) PH due to lung diseases and/or hypoxia, (4) chronic thromboembolic PH and other pulmonary artery obstructions, and (5) PH with unclear and/or multifactorial mechanisms. These groups differ hemodynamically regarding pre-capillary, post-capillary, or combined pathophysiology, with both PAH and Group 3 PH demonstrating pre-capillary PH [3]. 

The hallmark histopathological lesions in pre-capillary PH patients are medial hyperplasia and intimal fibrosis of the distal muscular arteries, loss and abnormal muscularization of small pre-capillary pulmonary arterioles, perivascular inflammation, and occurrence of plexiform vascular lesions [4]. Interestingly, plexiform pulmonary arterial lesions typical of pre-capillary PH were observed in human lung tissue from patients transplanted for advanced fibrotic interstitial lung disease (ILD), even in the absence of PH [5,6]. This indicates that these abnormalities may occur early during the development of Group 3 PH.

Treatment of PAH with vasodilator therapies targeting the prostacyclin, nitric oxide (NO), and endothelin pathways has significantly increased both survival and functional capacity. However, it should be noted that current treatment recommendations are based on clinical trials in patients with mPAP ≥25 mmHg, and further trials in patients are needed to define the benefit in those with mPAP between 20 and 25 mmHg.

In patients with idiopathic pulmonary fibrosis (IPF) and systemic sclerosis-associated interstitial lung disease (SSc-ILD), pulmonary hypertension (Group 3 PH) is a well-established risk factor for poor outcomes and increased morbidity. Treatment options are extremely limited, whereby there is only one licensed medicine for PH associated with lung diseases, inhaled treprostinil, which was recently shown to increase exercise ability in such patients [7]. A recent meta-analysis on the use of drugs approved for use in PAH demonstrated that they only provided small health-related quality-of-life benefits in patients with IPF and pulmonary hypertension. Furthermore, there was insufficient evidence to support their use [8], possibly with the exception of inhaled treprostinil. Thus, there is a high unmet need for novel therapeutic options with disease-modifying properties for PH associated with IPF and SSc-ILD.

The renin-angiotensin system (RAS) plays an important role in maintaining cardiovascular homeostasis, and there is substantial evidence supporting the involvement of RAS in the pathophysiology of PH [9,10,11]. Angiotensin II (Ang II) acts via two specific receptors, the angiotensin II type 1 receptor (AT_1_R) and the angiotensin II type 2 receptor (AT_2_R). The AT_1_R is ubiquitously expressed, while the expression of AT_2_R is normally low in adult tissues but can be upregulated during repair and regeneration [12,13]. The AT_1_R is mainly involved in blood pressure regulation through several different mechanisms related to vasoconstriction and fluid retention. The inducible AT_2_R, on the other hand, could be seen as a mechanism involved in the resolution of immune and vascular reactions to injury [14]. With regard to vascular effects, AT_2_R agonists have been reported to cause subtle arterial relaxation in vitro; however, per se, they do not generally lower systemic blood pressure in vivo [15]. Such a lack of translation into antihypertensive effects of AT_2_R agonists may depend on overriding vasoconstrictive AT_1_R activity. This is supported by the finding that infusion of the selective AT_2_R agonist CGP42112 failed to reduce blood pressure in spontaneously hypertensive rats unless administered in the presence of a low dose of the AT_1_R antagonist candesartan [16].

A role of the RAS and the AT2R in PAH/PH is also supported by findings that activation of angiotensin-converting enzyme-2 (ACE2), which catalyzes the production of Ang-(1-7) and Ang-(1-7) per se ameliorate experimental pulmonary hypertension in the rat [17,18,19,20]. Interestingly, while Ang-(1-7) is considered to signal mainly via the Mas receptor [21], it is also an AT_2_R agonist at nanomolar concentrations [22]. C21 (also known as Compound **21** or buloxibutid), the first AT_2_R agonist in clinical development, is a low molecular weight, orally available, selective, high-affinity AT_2_R agonist with low affinity to a range of other receptors tested, including the AT_1_R and the Mas receptor [23,24]. The unique signaling pathways and molecular mechanisms of AT_2_R activation are described in a recent comprehensive review [23]. In a phase 2 clinical trial, C21 decreased the need for oxygen therapy in hospitalized patients with COVID-19 [25], and the compound is currently also in an idiopathic pulmonary fibrosis clinical study [26]. Preclinical studies have demonstrated that C21 reduces PH in rodent models of pulmonary hypertension induced by monocrotaline [27] or pulmonary fibrosis and hypertension induced by bleomycin [28]. Importantly, it has also been shown that the AT_2_R is expressed in the rat lung and that C21 significantly increases this expression in both normal rats and in rats with PH after the monocrotaline challenge [27]. 

The purpose of the current study was to evaluate the efficacy of peroral C21 in pulmonary hypertension induced by Sugen 5416 and hypoxia, the SuHx-PH model. In this model, the animals develop a pathology resembling human PH, including the formation of plexiform lesions which are not seen in other frequently used animal models of pulmonary hypertension (e.g., chronic hypoxic and monocrotaline-injected rodents) [29]. This model shares similarities with both PAH and Group 3 PH [30].

## 2. Results

### 2.1. Hemodynamic Assessments by Echocardiography on Day 0, Day 21, and Day 56

**Figure 1:**
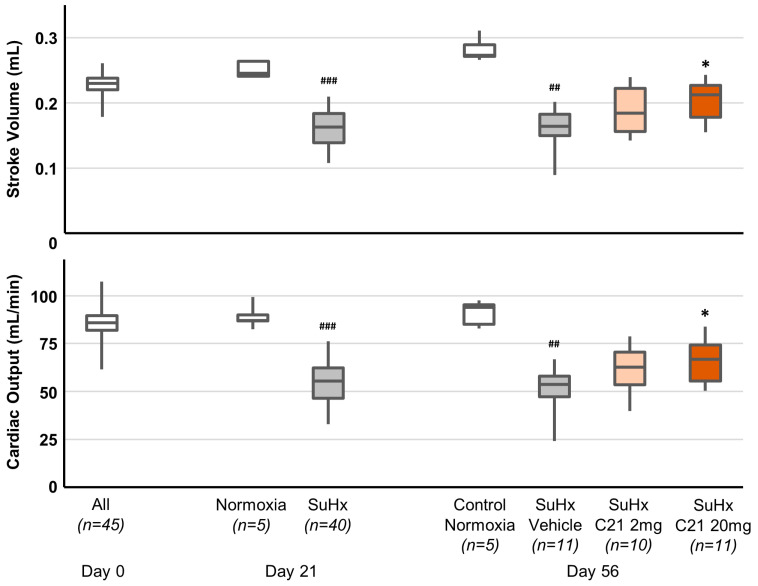
Hemodynamic assessments by echocardiography on day 0, day 21, and day 56. The box and whiskers represent the median, interquartile range, minimum and maximum values. ^##^ *p* < 0.01 and ^###^ *p* < 0.001 compared with normoxic animals (Mann–Whitney U-test). * *p* < 0.05 compared with SuHx Vehicle animals (Kruskal–Wallis test).

A single injection of Sugen 5416 followed by hypoxia (FiO_2_ = 0.10) (SuHx) resulted in profound hemodynamic changes on day 21, and the effects remained following normoxic conditions until day 56 (Figure 1, Table A1). Thus, right ventricle wall thickness (RVWT) increased significantly, and pulmonary artery maximum velocity (Vmax), pulmonary artery acceleration time (PAAT), cardiac output (CO), and stroke volume (SV) were significantly decreased. SuHx animals treated with C21 20 mg/kg from day 21 to day 55 demonstrated significantly improved CO and SV compared to animals treated with vehicle (Figure 1, Table A1). Animals treated with C21 2 mg/kg improved in a similar way, but there was no statistically significant difference compared to animals treated with vehicle or C21 20 mg/kg. 

### 2.2. Hemodynamic Assessments by Catheterization on Day 56

**Figure 2:**
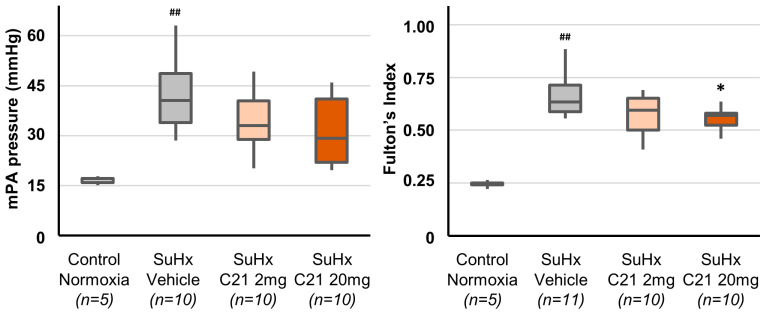
Mean pulmonary artery (mPA) pressure and right ventricular hypertrophy (Fulton’s Index) on day 56. The box and whiskers represent the median, interquartile range, minimum and maximum values. ^##^ *p* < 0.01 compared with normoxic animals (Mann–Whitney U-test). * *p* < 0.05 compared with SuHx Vehicle animals (Kruskal–Wallis test).

Hemodynamic assessments by catheterization on day 56 were performed one day after cessation of treatment when C21 was no longer present in the circulation, i.e., direct acute effects of C21 can thus be ruled out. Exposure to SuHx significantly increased right ventricular and pulmonary artery pressure and decreased saturation (Table A2). In animals treated with C21, there was a trend towards improvement in hemodynamics, including a decrease in mean pulmonary artery pressure, but this was not statistically significant, and there were no significant differences between animals treated with 2 or 20 mg/kg C21 (Figure 2, Table A2).

In post hoc analyses merging the two C21 groups, animals treated with C21 showed lower right ventricular and pulmonary artery pressures than the vehicle-treated animals, while there were no differences in systemic arterial blood pressure or heart rate. Furthermore, treatment with C21 (merged groups) increased saturation compared with the SuHx-vehicle group (Table A2). 

### 2.3. Right Ventricle Hypertrophy

Exposure to SuHx-induced right ventricular hypertrophy, measured as the weight ratio between the right ventricle and left ventricle, including septum (Fulton’s index), which was significantly increased in the SuHx-vehicle group compared to the control group (Figure 2). Further, right ventricle collagen content was significantly increased in the SuHx-vehicle group compared to the normoxic control group (Table A4).

Treatment with C21 20 mg/kg significantly decreased right ventricular hypertrophy as assessed by Fulton’s index, and a similar, although not statistically significant, trend was observed after treatment with C21 2 mg/kg, and there were no significant differences between the two C21 treated groups (Figure 2). Positive effects on right ventricular hypertrophy were supported in the post hoc analyses merging the two C21 groups (Table A4). Treatment with C21 tended to reduce the right ventricle collagen content compared with the SuHx Vehicle group, but the differences were not statistically significant (Table A4).

Within all SuHx exposed groups combined, the level of right ventricular hypertrophy was closely correlated with the histopathological signs of vascular remodeling and hemodynamic outcomes (Table A5).

### 2.4. Vascular Remodeling

**Figure 3:**
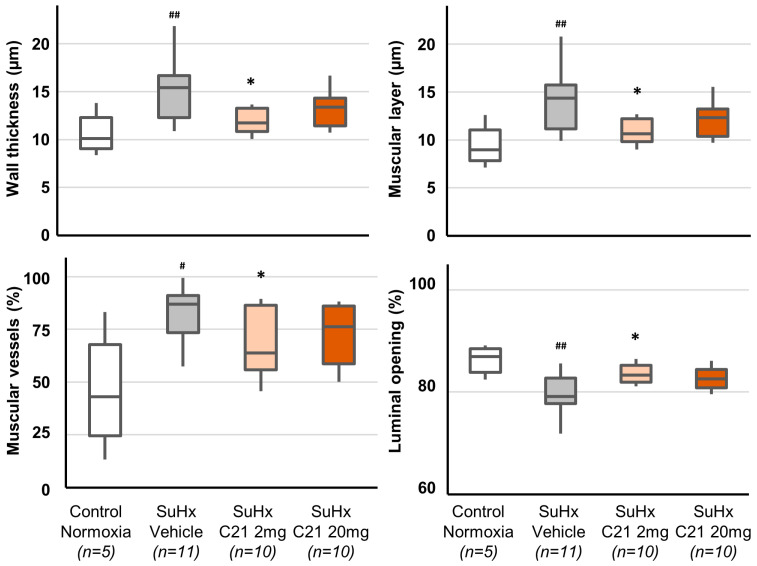
Wall morphology in vessels >100 μm. The box and whiskers represent the median, interquartile range, minimum and maximum values. ^#^ *p* < 0.05 and ^##^ *p* < 0.01 compared with normoxic animals (Mann–Whitney U-test). * *p* < 0.05 compared with SuHx Vehicle animals (Kruskal–Wallis test).

The results confirm that the rat SuHx pulmonary hypertension model displays marked endothelial proliferation reminiscent of human disease. Thus, exposure to SuHx induced vascular remodeling with increased area stained for von Willebrand Factor (vWF; endothelial cell marker) and muscular portions of the vessel walls and vascular remodeling (Figure 3, Table A3).

SuHx-exposed animals treated with C21 2 mg/kg showed, compared to vehicle-treated animals, significantly reduced thickening of the vascular wall, in particular the muscular layer, proportion of muscular vessels, and increased luminal opening in large vessels (Figure 3, Table A3). Treatment with C21 20 mg/kg significantly reduced the vWF area, increased the proportion of non-obliterated medium-size vessels, and reduced the proportion of semi-obliterated medium-size vessels. There were no significant differences in any parameters reflecting vascular remodeling between animals treated with 2 or 20 mg/kg C21 (Table A3). However, in post hoc analyses where a merged C21 treatment group (2 and 20 mg/kg combined) was compared with vehicle treatment, vessels of all sizes showed a decreased thickness of the vessel wall and the muscular layer, increased luminal opening, and increased the proportion of non-obliterated vessels.

### 2.5. Collagen Deposition and Dense Areas in the Lungs

**Figure 4:**
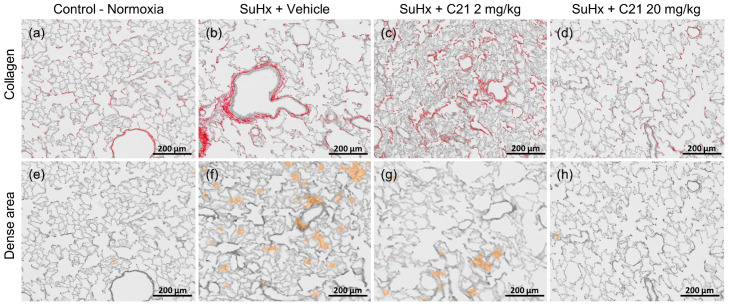
Representative images for fibrosis and collagen deposition and dense area in the lung of rats exposed to SuHx and C21. Areas with collagen deposition identified by picrosirius red staining (**a**–**d**). Dense areas containing fibroblasts, myofibroblasts, and newly formed collagen were identified by automated morphometric analysis (**e**–**h**).

In vehicle-treated animals, the SuHx challenge was associated with increased fibrillar collagen content and dense areas containing fibroblasts, myofibroblasts, and newly formed collagen in the lung, both of which were significantly reduced by the 20 mg/kg dose of C21 but not by the 2 mg/kg dose (Table 1, Figure 4). Lung hydroxyproline was not significantly increased after SuHx and was not affected by C21 treatment (Table 1).

## 3. Discussion

This study demonstrated that the selective AT_2_R agonist C21 decreased vascular remodeling and improved the hemodynamics in rats with pulmonary hypertension induced by Sugen 5416 and hypoxia (SuHx), a model with histopathological features that resemble human PAH and Group 3 PH [30]. Importantly, the model evaluated therapeutic rather than preventive (prophylactic) efficacy, as treatment with C21 was started after the animals had developed pulmonary hypertension. Further, because C21 has a relatively short half-life and was not administrated on the day of sacrifice, the hemodynamic effects are not due to the direct acute vascular effects of C21 but rather to the reduced remodeling. Moreover, the study showed that C21 reduced the SuHx-induced pulmonary collagen deposition supporting the relevance for clinical conditions where pre-capillary pulmonary hypertension is associated with fibrotic lung disease, e.g., group 3 PH and connective tissue disease. 

The apparent lack of clear dose-dependency regarding vascular or hemodynamic effects of C21 suggests that, for several of the measured parameters, a “ceiling effect” was reached with the lower dose, which results in a free C21 plasma in the range of the IC_50_ in previous AT_2_R binding studies. Interestingly, and in contrast to the cardiovascular effects, C21 dose-dependently reduced pulmonary collagen deposition. The reason for this difference is not known, but one possible explanation is cellular/tissue differences in AT_2_R expression. Based on the absence of dose dependency regarding vascular and hemodynamic effects, exploratory post hoc analyses were performed on these parameters (see below). 

Treatment with C21 reduced vascular remodeling in the rat SuHx model as demonstrated by decreased endothelial proliferation in animals treated with C21 20 mg/kg, reduced thickening of the vascular wall, in particular the muscular layer, and decreased proportion of muscularized vessels >100 μm in animals treated with C21 2 mg/kg. In the post hoc analyses, similar results were obtained for vessels of all sizes, and there was also a decrease in vessel obliteration. As a result, the diastolic pulmonary artery pressure, a sensitive indicator of efficacy in pulmonary hypertension hemodynamics, and right ventricular pressure were reduced in the combined C21 groups. The apparent reduction of systolic pulmonary artery pressure cannot be a consequence of a failing right ventricle because stroke volume, V_max_, and cardiac output increased. This suggests that the effect of C21 on pulmonary artery pressure is a result of reduced resistance in the pulmonary vascular bed, leading to decreased demand on the right ventricle and reduced right ventricle hypertrophy. The latter is supported by the significant reduction in Fulton’s index, which correlated closely with the histopathological signs of vascular remodeling and hemodynamic outcomes.

The mechanism by which C21 reduced pulmonary vascular remodeling and improved the hemodynamics could be either chronic vasodilation or a direct effect on remodeling processes. Although C21 has been shown to have vasodilating effects in vitro, C21 does not generally lower systemic blood pressure in vivo [15]. On the other hand, and supporting direct effects on the remodeling process, C21 has previously been demonstrated to reduce cardiopulmonary remodeling and pulmonary hypertension in animals exposed to bleomycin [28] or monocrotaline [27]. In line with these previous studies of pulmonary fibrosis/hypertension, C21 also reduced the increase in pulmonary collagen deposition in the SuHx model.

C21 has previously shown efficacy in rat models of monocrotaline and bleomycin-induced pulmonary hypertension. However, it is difficult to compare models with different methods to induce PH, and it is well established that monocrotaline and bleomycin are more acute and more proinflammatory than Sugen plus hypoxia. Further, in the previous studies, C21 treatment was initiated 3 days (bleomycin) or 2 weeks (monocrotaline) after pulmonary challenge, while in the current study, treatment was started 3 weeks after initiation of the pulmonary pathology. Moreover, in the previous studies, C21 was injected intraperitoneally without measurements of plasma levels of C21, another factor that makes it difficult to compare the results.

The SuHx-induced increase of dense areas in the lung parenchyma with elevated collagen content is similar to key features of human PAH and Group 3 pulmonary hypertension [31]. Moreover, pulmonary hypertension in the SuHx model is induced by a combination of hypoxic vasoconstriction and an induced imbalance between angiogenic and angiostatic factors, which resembles the pathogenesis of pulmonary hypertension in IPF [32]. 

Animals in SuHx studies progress through an initial phase of pulmonary vasoconstriction with limited inflammation, followed by normoxic vascular remodeling, during which pulmonary endothelial and smooth muscle cells proliferate to increase vessel wall thickness. Fibrosis, as measured by collagen deposition and the presence of histological fibrosis with increased alveolar wall and peri-bronchial collagen staining in the lungs, thus appears in the later phase of SuHx-induced pulmonary hypertension. These changes are reminiscent of non-specific interstitial pneumonia (NSIP) seen in connective tissue disease and highlight the particular relevance of our findings to connective tissue-associated pre-capillary PH. Therefore, the observed effect of C21 on adventitial fibrosis and associated lung fibrosis is particularly interesting in interstitial lung diseases with associated pulmonary hypertension. It is interesting that similar observations with reduced pulmonary fibrosis and vasculopathy were also seen in a Sugen-dependent transgenic mouse model of scleroderma-associated pulmonary hypertension using a PPAR agonist with antifibrotic properties [33].

Based on the studies using the bleomycin and the monocrotaline models [27,28] and a recent review of the unique signaling pathways and molecular mechanisms of AT_2_R activation [23], the beneficial effects of C21 treatment are believed to be mediated by reduced levels of pro-fibrotic grow factors and proinflammatory cytokines such as TGF-β, CTGF, IL-1β, IL-6, and TNF.

The main purpose of this study was to gain further support for performing a clinical trial with an AT_2_R agonist in pulmonary hypertension (which is now being planned). 

Pulmonary hypertension is a common complication of interstitial lung disease (ILD) and is associated with high morbidity and increased mortality due to decreased pulmonary blood flow and poor gas exchange, leading to compromised cardiac function. Treatment options are limited, and it has been reported that vasodilator agents approved for PAH are less effective in this patient group. Further, due to the non-homogeneous fibrotic pattern in ILD, vasodilatory treatment may increase a mismatch between ventilation and perfusion, which may even have deleterious effects. Despite this, a recent meta-analysis of randomized control trials of therapies approved for PAH in patients with CTD-PH demonstrated a reduction in the risk of morbidity and mortality [34,35], indicating that a carefully supervised vasodilatory treatment could be beneficial. However, treatment with vasodilators does not seem to prevent the development of pulmonary hypertension since a vast majority of patients with systemic sclerosis receive vasodilating/vasoactive therapy for their Raynaud’s phenomenon and digital ulcers [36]. However, a high proportion still develops pulmonary hypertension [37]. Thus, there is a significant medical need for novel therapies addressing the structural and perivascular fibrotic processes determining pulmonary hypertension in the context of ILD and for pathways where additive benefit can be seen on top of current therapies.

In conclusion, the effects of C21 on remodeling, hemodynamic alterations, and increased pulmonary collagen deposition in the Sugen-Hypoxia model suggest that treatment with an AT_2_R agonist may have a place not only in PAH but also in the much-needed treatment of Group 3 pulmonary hypertension associated with ILD, or in cases of systemic sclerosis and other connective tissue diseases in which Group 1 pulmonary hypertension develops in the context of concurrent lung fibrosis.

## 4. Materials and Methods

### 4.1. Animals and Treatments

**Figure 5:**
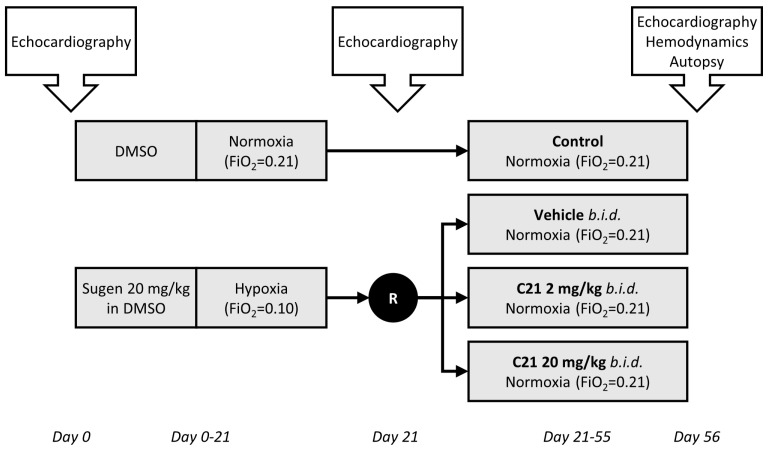
Overview of the study design. Animals received a single subcutaneous injection of Sugen (20 mg/kg) in 2 mL 100% DMSO and were kept under hypoxic conditions for 21 days (SuHx groups). On day 21, an echocardiogram was performed carried out to achieve an even distribution of the disease in the three study groups. The animals were exposed to ambient oxygen levels from day 22 to day 56. From day 21 to day 55, C21 (2 mg/kg or 20 mg/kg) or vehicle was administered twice a day by gavage. Control group animals received a single subcutaneous injection of 2 mL 100% DMSO on day 0 and were exposed to ambient oxygen for the total study period.

Male Sprague–Dawley rats (Charles River Laboratories, St-Constant, QC, Canada) aged 7–9 weeks and with an initial weight between 200 and 250 g were pair-housed in a temperature- (23 °C) and humidity- (65%) controlled housing room on a 12-h light/dark cycle and had ad libitum access to standard rat chow and water for the duration of the study. After an acclimation period of 5 days, 5 animals received one subcutaneous injection of 2 mL 100% DMSO and were exposed to ambient oxygen levels for the total study period of 56 days (control group). Another group of animals received a single subcutaneous injection of Sugen (20 mg/kg) in 2 mL 100% DMSO and were placed in cages for which the controlled air was adjusted to receive a FiO_2_ equivalent to 0.10 (10%) using a mixture of nitrogen and ambient air. They were kept under these hypoxic conditions for 21 days (SuHx group). While in hypoxia, cages were cleaned and changed once a week, exposing the animals to ambient oxygen levels for less than 10 min in total. On day 21, when the animals had developed functional symptoms of PH (Figure 1), an echocardiogram was carried out under light isoflurane sedation (1–1.5% in ambient air), and the results were used to randomize the animals across three treatment groups (*n* = 10–11 in each) to achieve an even distribution of the disease. Animals within the same treatment group were pair-housed and exposed to ambient oxygen levels (21%) from day 22 to day 56. From day 21 to day 55, C21 (2 mg/kg or 20 mg/kg in sodium carbonate buffer) or sodium carbonate buffer alone (SuHx-vehicle group) was administered twice a day by gavage in a volume of 10 mL/kg of body weight (Figure 5). 

During the preclinical development of C21 prior to the completed and ongoing clinical studies, the pharmacokinetics of these two oral doses of C21 were studied in male Sprague–Dawley rats. Briefly, taking into account the known high degree (99%) of plasma protein binding of C21 in the rat, the peak unbound/free plasma concentration of C21 was approximately 5 and 80 nM after the 2 and 20 mg/kg dose, respectively, with a plasma half-life in the 2 h range [24]. For comparison, in multiple AT_2_R binding studies, the IC_50_ for C21 has been found to be 2–5 nM [22,24]. The rats were weighed before dose administration, and dose volumes were calculated based on body weight. Rats were weighed once every 3 days, and the dose volume was adjusted to achieve the correct dose level. 

### 4.2. Hemodynamic Measurements

At the end of the experiment (day 56), an echocardiogram was carried out under light isoflurane sedation (1–1.5% in ambient air) to measure the pulmonary artery maximum velocity (V_max_) and velocity time integral, the pulmonary artery diameter, and the heart rate, from which cardiac output and stroke volume were calculated. The animals were rapidly transferred to the hemodynamic suite, where the rats were anesthetized with a mixture of 2 to 2.5% isoflurane USP (Abbot Laboratories, Montreal, QC, Canada) in oxygen. The absence of nociception was monitored using changes in heart rate in response to tactile stimulation and pressure. Hemodynamic and functional parameters were recorded continuously for 5 min or until loss of pulmonary arterial pressure signal, whichever came first. Blood oxygen saturation (SO_2_) was recorded with a pulse oximeter (Nonin, Plymouth, MN, USA) attached to the left front paw of the animal. Arterial systemic pressure was recorded with a fluid-filled catheter (AD Instruments, Colorado Springs, CO, USA) inserted into the right upper femoral artery. Right ventricular and pulmonary arterial blood pressures were recorded by inserting a solid-state, tip-mounted pressure transducer (Millar, Houston, TX, USA) through the apex of the right ventricle and pushing the transducer past the pulmonary valve into the pulmonary artery. The systolic, diastolic, and mean pressures were measured in mmHg post-acquisition. Mean ventricular and pulmonary blood pressure values were calculated using the following formula:

(1) Mean Pressure=Systolic pressure+(Systolic pressure−Diastolic pressure)/3 

### 4.3. Tissue Sampling and Histology

The anesthetized rats were then exsanguinated under deep isoflurane anesthesia (1.5–2.0% isoflurane vaporized in 100% oxygen at a flow of 1 L/min, with heart rate used as an indicator of anesthetic depth. Isoflurane was adjusted to achieve a heart rate of 280–300 bpm) as per the recommendations of IPST’s veterinarian and IACUC guidelines. The pulmonary circulation and the heart were flushed after the lungs-heart had been harvested from the animal and mounted onto a gravity-driven perfusion system. At least 15 mL of saline solution (0.9% NaCl) was used for flushing out the blood. The left lung was fixed by arterial and airway perfusion at pathophysiological perfusion pressures with 10% NBF and embedded in paraffin. The upper lobe of the right lung was cut, snap-frozen, and stored at −80 °C, for quantification of hydroxyproline. The cardiac tissue was excised to measure the wet weights of the right ventricle and left ventricle, including the septum, as part of the Fulton index. The right ventricle was snap-frozen and stored at −80 °C for quantification of collagen using Image J color detection thresholds following picrosirius red staining of the fixed heart.

(2) Fulton’s Index=(Right ventricle weight)/(Left ventricle+Septum weight)

For each animal, 2 slides of 2 sections (5 μm thick) from the formalin-fixed paraffin-embedded blocks were cut transversally and spaced by 50 μm. Two sections were laid on the same slide and immune-stained for von Willebrand factor (vWF; polyclonal rabbit antibody from Abcam (Cambridge, UK) at 1/2000 dilution with 20 min incubation at 35 °C; secondary FITC mouse anti-rabbit monoclonal antibody from Abcam was used at a dilution of 1:1000). The slides were incubated for 1 h in the dark, at room temperature, and the other two sections were laid on a second slide and stained for picrosirius red (PSR) according to an earlier described method [38]. All stained slides were then scanned using the Hamamatsu 2.0 HT scanner at 20x magnification (0.452 μm per pixel) for automatic quantification by MorphoQuant™ (Biocellvia, Marseille, France). Pulmonary high-density tissue corresponding to fibrous thickening of alveolar parenchyma and expressed as “Area of increased collagen deposition (% of lung section area),” pulmonary collagen area expressed as “Collagen (% of lung section area)” were quantified as described before [39,40]. The von Willebrand factor area was used as a marker of endothelial cells. The label was intense in endothelial cells but spread out through the muscle layer until complete fading in the adventitia. The automated detection of blood vessels was based on the recognition of empty spaces surrounded by vWF labeling. The intense vWF label was assimilated to the endothelial layer and expressed as “vWF area (% of lung section area),” and the smooth muscle layer was the layer with less intense labeling.

Each blood vessel was then assimilated into a perfectly circular circle for subsequent measurements for simplification. Blood vessels were categorized according to their diameter (defined as the other outer limit of the smooth muscle layer) into three classes: small (S), medium (M), and large (L) vessels (≤50; >50–100; >100 μm, respectively). For each category, the thickness of the vessel wall, the endothelial (vWF strongly positive), and muscular layers was assessed (vWF lightly positive), and the proportion of muscular vessels was calculated. Further, the luminal opening (ratio of the lumen to the blood vessel diameter) was assessed, as well as the proportion of obliterated (luminal opening <45%), semi-obliterated (luminal opening ≥45% and <60%), and non-obliterated vessels (luminal opening ≥60%) vessels.

### 4.4. Quantification of Lung Hydroxyproline Content

Hydroxyproline in the right lung was quantified using a commercial kit from Abcam (Ab222941) used as per the manufacturer’s instructions. The right lobes were homogenized in PBS and hydrolyzed in 10 N NaOH for 1 h at 120 °C. Then, the basic suspension was neutralized using 10 N HCl and centrifuged to generate a clear supernatant. The hydroxyproline concentration was determined by the reaction of oxidized hydroxyproline with 4-(Dimethylamino) benzaldehyde (DMAB), measured on a Molecular Devices (San Jose, CA, USA) SpectraMax plate reader at 560 nm. The total amount of hydroxyproline was calculated based on the right lobe weight.

### 4.5. Statistics

Due to the explorative nature of the study with no predefined primary endpoint, no power calculation was performed, and the study size was based on experience from multiple similarly designed studies performed at the same testing facility. Since the Anderson-Darling test could not confirm normal distribution for a high proportion of the variables, significance was tested by non-parametric tests. As an initial step, the SuHx-vehicle group was compared with the control group to demonstrate assay sensitivity using the Mann–Whitney U-test). Thereafter, the three SuHx groups were compared with each other by Kruskal–Wallis test. There were no significant differences or consistent tendency on vascular remodeling or hemodynamic assessments between the two C21 dose levels, which both result in a peak exposure at or well above IC_50_ for the AT_2_R. Thus, an exploratory post hoc analysis was performed comparing the merged SuHx-C21 group with the SuHx-vehicle groups tested using the Mann–Whitney U-test. Correlations were analyzed using Spearman’s rank correlation test. All statistical analyses were performed on SPC for Excel (Version 6.0.1.1). Statistical significance was defined as *p* < 0.05.

## Figures and Tables

**Table 1:** Collagen, dense area, and hydroxyproline in the lungs of rats exposed to Sugen-hypoxia.

	Control Normoxia (*n* = 5)	SuHx Vehicle (*n* = 11)	SuHx C21 2 mg/kg (*n* = 10)	SuHx C21 20 mg/kg (*n* = 10)
Collagen content [%]	5.21 [5.08;5.57]	6.27 [5.75;7.34] ^#^	6.39 [6.04;7.22]	5.19 [4.92;5.41] **^,§§§^
Dense area [%]	2.63 [2.08;2.84]	4.81 [3.76;5.96] ^##^	4.65 [4.44;6.33]	3.01 [2.66;4.37] *^,§§^
Hydroxyproline [μm/mg tissue]	60 [51;74]	64 [57;81]	55 [51;68]	63 [58;70]

Values are median (interquartile range). **^#^** *p* < 0.05 and **^##^** *p* < 0.01 compared with the control group (Mann–Whitney U-test). * *p* < 0.05 and ** *p* < 0.01 compared with the vehicle group, and **^§§^** *p* < 0.01 and **^§§§^** *p* < 0.001 compared with the C21 2 mg/kg group (Kruskal–Wallis test). Dense areas containing fibroblasts, myofibroblasts, and newly formed collagen were identified by automated morphometric analysis. Hydroxyproline concentration was assessed as described in Material and Methods. Areas with collagen deposition were identified by picrosirius red staining. For a description of the SuHx model and experimental groups, see Figure 1.

## Data Availability

The data presented in this study are openly available in Dryad at https://doi.org/10.5061/dryad.9s4mw6mk1, published 9 April 2023.

## Appendix A

**Table A1:** Hemodynamic assessments by echocardiography on day 0, day 21, and Day 56.

Time	Group	RVWT (mm)	PAD (cm)	HR (bpm)	V_max_ (cm/s)	VTI (cm)	PAAT (ms)	ET (ms)	CO (mL/min)	SV (mL)
Day 0	All (*n* = 45)	0.44 [0.44;0.53]	0.24 [023;0.24]	377 [361;396]	84.5 [81.7;88.8]	5.20 [5.08;5.42]	29.6 [22.2;29.6]	81 [74;89]	85.9 [81.9;89.7]	0.23 [0.22;0.24]
Day 21	Control-Normoxia (*n* = 5)	0.50 [0.44;0.53]	0.24 [0.24;0.25]	355 [345;361]	95.8 [92.5;96.5]	5.42 [5.29;5.79]	29.6 [29.4;29.6]	81 [81;81]	87.1 [86.8;89.9]	0.25 [0.24;0.26]
	SuHx (*n* = 37)	1.13 ^###^ [0.98;1.20]	0.25 ^#^ [0.24;0.26]	338 [323;353]	61.3 ^###^ [58.0;66.5]	3.26 ^###^ [2.83;3.64]	18.4 ^##^ [14.8;22.2]	81 [77;84]	55.5 ^###^ [46.5;62.3]	0.16 ^###^ [0.14;0.18]
Day 56	Control-Normoxia (*n* = 5)	0.53 [0.53;0.53]	0.25 [0.25;0.25]	312 [306;338]	93.7 [92.3;95.1]	5.56 [5.52;5.86]	37.0 [37.0;37.0]	96 [89;96]	93.9 [85.1;95.2]	0.27 [0.27;0.29]
	SuHx and Vehicle (*n* = 11)	1.24 ^##^ [1.20;1.29]	0.25 [0.24;0.25]	306 [306;328]	66.2 ^##^ [60.5;71.5]	3.58 ^##^ [3.18;3.74]	22.2 ^##^ [22.2;22.2]	89 [81;89]	53.7 ^##^ [47.2;58.0]	0.16 ^##^ [0.15;0.18]
	SuHx and 2 mg/kg C21 (*n* = 10)	1.11 [0.98;1.31]	0.25 [0.25;0.26]	328 [301;343]	65.8 [57.7;68.8]	3.83 [3.21;4.44]	25.9 [16.6;29.6]	93 [89;96]	62.7 [53.6;70.5]	0.18 [0.16;0.22]
	SuHx and 20 mg/kg C21 (*n* = 11)	1.06 [1.02;1.38]	0.25 * [0.25;0.26]	325 [304;345]	71.1 [65.1;78.5]	4.01 [3.56;4.53]	22.2 [22.2;25.9]	89 [81;96]	66.8 * [55.5;74.3]	0.21 * [0.18:0.23]
	SuHx and All C21 (*n* = 21)	1.06 [0.98;1.33]	0.25 ^§^ [0.25;0.26]	325 [301;345]	68.3 [61.3;76.1]	4.01 [3.46;4.51]	22.2 [22.2;29.6]	89 [81;96]	65.5 ^§^ [54.6;73.7]	0.20 ^§^ [0.16;0.22]

RVWT: right ventricle wall thickness; PAD: pulmonary artery diameter; HR: heart rate; Vmax: pulmonary artery maximum velocity; VTI: right ventricular outflow tract velocity time integral; PAAT: pulmonary artery acceleration time; ET: ejection time; CO: cardiac output; SV: stroke volume. Values are median [interquartile range]. ^#^ *p* < 0.05, ^##^ *p* < 0.01 and ^###^ *p* < 0.001 compared with the control group (Mann–Whitney U-test). * *p* < 0.05 compared with the SuHx vehicle group (Kruskal–Wallis test). ^§^ *p* < 0.05 compared with the SuHx vehicle group in post hoc analyses (Mann–Whitney U-test).

**Table A2:** Hemodynamic assessments by catheterization on day 56.

	Control Normoxia (*n* = 5)	SuHx Vehicle (*n* = 11)	SuHx C21 2 mg/kg (*n* = 10)	SuHx C21 20 mg/kg (*n* = 10)	SuHx All C 21 (*n* = 20)
Saturation (%)	98 (96;98)	94 (91;96) ^#^	97 (94;97)	96 (93;98)	97 (94;98) ^§^
Arterial pressure (mmHg)					
Diastolic	95.5 [68.2;110.5]	93.5 [72.5;104.4]	104.2 [79.4;112.4]	103.2 [85.7;118.3]	103.2 [81.9;1116.1]
Systolic	126.9 [93.5;141.1]	116.9 [101.3;134.1]	133.5 [108.6;146.9]	130.9 [113.9;147.3]	130.9 [112.9;145.9]
Mean	106.0 [76.6;120.7]	102.0 [82.1;114.6]	114.6 [89.1;122.4]	112.9 [95.1;128.5]	112.9 [92.3;126.1]
Right ventricular pressure (mmHg)					
Diastolic	3.8 [3.6:5.1]	4.3 [3.2;5.2]	2.5 [1.5;4.0]	2.4 [1.3;4.0]	2.5 [1.4;3.9] ^§^
Systolic	22.4 [21.3;24.5]	62.0 [46.3;82.3] ^##^	50.3 [43.7;58.9]	48.4 [32.3;63.0]	49.5 [41.0;61.4]
Mean	10.3 [9.9;11.0]	24.0 [18.3;30.4] ^##^	18.8 [16.1;22.0]	17.4 [12.0;24.8]	17.9 [14.4;22.9] ^§^
Pulmonary artery pressure (mmHg)					
Diastolic	13.4 [12.4;14.2]	28.4 [24.5;33.3] ^##^	24.7 [17.1;31.7]	20.1 [14.1;30.7]	22.6 [15.3;31.0] ^§^
Systolic	23.2 [21.7;24.8]	65.0 [47.2;83.9] ^##^	51.6 [44.0;61.6]	47.3 [32.6;62.8]	49.0 [41.7;61.8]
Mean	17.1 [15.5;17.5]	40.6 [32.8;50.0] ^##^	33.0 [26.9;42.5]	29.2 [21.4;41.7]	30.5 [22.3;41.9] ^§^

Values are median [interquartile range]. ^#^ *p* < 0.05 and ^##^ *p* < 0.01 compared with the control group (Mann–Whitney U-test). ^§^ *p* < 0.05 and compared with the vehicle group in post hoc analyses (Mann–Whitney U-test).

**Table A3:** Assessments of vasculopathy in the lungs of rats exposed to Sugen-hypoxia.

	Control Normoxia (*n* = 5)	SuHx Vehicle (*n* = 11)	SuHx C21 2 mg/kg (*n* = 10)	SuHx C21 20 mg/kg (*n* = 10)	SuHx All C21 (*n* = 20)
vWF Area (%)	1.22 [1.17;1.72]	1.94 [1.87;2.62] ^##^	1.90 [1.76;2.03]	1.75 [1.69;1.89] *	1.85 [1.70;2.03]
Vessel Wall (µm)					
Small vessels	5.87 [5.00;5.98]	7.40 [6.78;7.76] ^##^	6.88 [6.44;7.10]	7.01 [6.41;7.31]	6.93 [6.46;7.21] ^§^
Medium vessels	8.20 [7.93;9.40]	12.17 [10.84;13.15] ^##^	10.99 [10.34;11.47]	11.15 [10.52;12.13]	10.99 [10.47;12.01] ^§^
Large vessels	10.11 [9.05;12.28]	15.42 [12.28;16.68] ^##^	11.75 [10.83;13.28] *	13.39 [11.42;14.33]	12.42 [10.92;13.68] ^§^
Endothelial Layer (µm)					
Small vessels	0.88 [0.86;0.90]	0.79 [0.78;0.85] ^##^	0.82 [0.78;0.84]	0.83 [0.80;0.87]	0.83 [0.80;0.86]
Medium vessels	1.03 [0.96;1.07]	0.84 [0.82;0.87] ^##^	0.86 [0.82;0.89]	0.90 [0.85;0.96]	0.88 [0.83;0.91]
Large vessels	1.23 [1.19;1.23]	1.05 [1.00;1.08] ^##^	1.06 [1.00;1.12]	1.07 [1.03;1.14]	1,06 [1.03;1.13]
Muscular Layer (µm)					
Small vessels	4.98 [4.13;5.09]	6.65 [5.92;6.98] ^##^	6.05 [5.63;6.29]	6.13 [5.59;6.51]	6.06 [5.64;6.41] ^§^
Medium vessels	7.25 [6.89;8.38]	11.33 [10.02;12.32] ^##^	10.15 [9.45;10.66]	10.22 [9.56;11.30]	10.15 [9.56;11.16] ^§^
Large vessels	8.95 [7.82;11.06]	14.37 [11.14;15.75] ^##^	10.66 [9.83;12.22] *	12.32 [10.38;13.24]	11.33 [9.88;12.64] ^§§^
Muscular Vessels (%)					
Small vessels	1.2 [0.4;2.0]	17.6 [12.6;22.3] ^##^	13.3 [10.9;16.3]	15.1 [10.2;17.7]	14.1 [11.2;17.0] ^§^
Medium vessels	11.1 [8.2;33.3]	65.9 [54.7;74.2] ^##^	56.4 [50.8;62.3]	63.1 [53.9;72.7]	57.9 [52.0;740.5]
Large vessels	43.1 [24.5;67.8]	87.0 [73.4;91.1] ^#^	63.9 [55.9;86.3] *	76.2 [58.7;86.0]	65.2 [57.7;85.7] ^§^
Luminal Opening (%)					
Small vessels	65.5 [65.0;69.5]	58.4 [56.8;61.1] ^##^	60.6 [59.6;62.3]	59.9 [58.4;62.6]	60.2 [59.1;62.1] ^§^
Medium vessels	75.5 [72.4;76.8]	65.5 [62.1;67.2] ^##^	67.9 [66.7;69.2]	67.6 [65.2;69.3]	67.9 [65.6;69.0] ^§^
Large vessels	86.9 [83.9;88.4]	79.1 [77.8;82.8] ^##^	83.3 [81.9;85.2] *	82.6 [80.8;84.4]	82.9 [81.5;85.0] ^§§^
Non-obliterated Vessels (%)					
Small vessels	80.0 [76.2;91.8]	43.5 [36.1;55.8] ^##^	54.1 [49.1;59.5]	51.1 [46.6;64.7]	52.1 [47.5;62.1] ^§^
Medium vessels	98.9 [94.2;99.8]	68.7 [59.4;72.8] ^##^	76.3 [72.7;80.6]	77.7 [72.5;84.3] *	76.8 [72.9;81.6] ^§§^
Large vessels	100.0 [100.0;100.0]	98.8 [94.3;100.0]	99.6 [98.7;100.0]	100.0 [99.1;100.0]	100 [99.1;100.0]
Semi-obliterated Vessels (%)					
Small vessels	19.5 [8.2;23.0]	42.0 [37.5;52.7] ^##^	39.2 [34.4;42.1]	40.6 [29.7;46.4]	40.3 [32.6;43.7]
Medium vessels	1.1 [0.2;5.3]	23.4 [19.8;27.8] ^##^	17.2 [15.4;20.4]	15.6 [12.5;20.3] *	17.2 [14.0;19.6] ^§§^
Large vessels	0.0 [0.0;0.0]	1.2 [0.0;3.9]	0.4 [0.0;1.3]	0.0 [0.0;0.9]	0.0 [0.0;0.9]
Obliterated Vessels (%)					
Small vessels	0.4 [0.0;0.8]	8.8 [6.7;12.3] ^##^	6.4 [5.8;9.5]	7.9 [4.3;10.0]	7.1 [5.4:9.8]
Medium vessels	0.0 [0.0;0.4]	7.1 [6.0;11.6] ^##^	5.0 [4.0;9.3]	4.0 [3.1;6.5]	4.7 [3.4;8.1] ^§^
Large vessels	0.0 [0.0;0.0]	0.0 [0.0;0.0]	0.0 [0.0;0.0]	0.0 [0.0;0.0]	0.0 [0.0;0.0]

Values are median [interquartile range]. ^#^ *p* < 0.05 and ^##^ *p* < 0.01 compared with the control group (Mann–Whitney U-test). * *p* < 0.05 compared with the vehicle group (Kruskal–Wallis test). ^§^ *p* < 0.05 and ^§§^ *p* < 0.01 compared with the vehicle group in post hoc analyses (Mann–Whitney U-test).

**Table A4:** Right ventricle hypertrophy.

	Control Normoxia (*n* = 5)	SuHx Vehicle (*n* = 11)	SuHx C21 2 mg/kg (*n* = 10)	SuHx C21 20 mg/kg (*n* = 10)	SuHx All C 21 (*n* = 20)
Fulton’s index	0.24 [0.23;0.26]	0.63 [0.58;0.72] ^##^	0.60 [0.49;0.65]	0.57 [0.51;0.59] *	0.57 [0.50;0.64] ^§^
Left ventricular weight (g)	1.14 [1.03;1.28]	1.09 [1.01;1.20]	1.20 [1.06;1.32]	1.16 [1.04;1.19]	1.17 [1.05;1.23]
Right ventricular weight (g)	0.28 [0.26;0.30]	0.74 [0.64;0.81] ^##^	0.67 [0.59;0.75]	0.59 [0.54;0.74]	0.63 [0.56;0.74]
Right ventricle collagen (mg)	2.06 [1.79;2.24]	4.90 [3.66;5.31] ^##^	4.18 [3.72;4.76]	4.05 [3.28;5.20]	4.18 [3.72;4.76]

Values are median [interquartile range]. ^##^ *p* < 0.01 compared with the control group (Mann–Whitney U-test). * *p* < 0.05 compared with the vehicle group (Kruskal–Wallis test). ^§^ *p* < 0.05 compared with the vehicle group in post hoc analyses (Mann–Whitney U-test).

**Table A5:** Correlation between right ventricular hypertrophy (Fulton’s index) and histopathological signs on vascular remodeling and hemodynamic parameters in animals exposed to SuHx and treated with vehicle or C21.

Vascular Remodeling	rho	Hemodynamic Parameters	rho
VWF Area (%)	0.411 *	Saturation (%)	−0.299
Vessel wall thickness (µm)		Systemic arterial pressure (mmHg)	
Small vessels	0.434 *	Diastolic	−0.163
Medium vessels	0.480 **	Systolic	−0.237
Large vessels	0.432 *	Mean	−0.209
Endothelial layer (µm)		Right ventricular pressure (mmHg)	
Small vessels	−0.204	Diastolic	0.341
Medium vessels	−0.292	Systolic	0.455 *
Large vessels	−0.193	Mean	0.501 **
Muscle layer (µm)		Pulmonary artery pressure (mmHg)	
Small vessels	0.450 *	Diastolic	0.475 **
Medium vessels	0.504 **	Systolic	0.439 *
Large vessels	0.444 *	Mean	0.485 **
Luminal opening (%)		Stroke volume (mL)	−0.560 **
Small vessels	−0.477 **	V_max_ (cm/s)	−0.630 ***
Medium vessels	−0.529 **	Cardiac output (mL/min)	−0.537 **
Large vessels	−0.471 **	Stroke volume (mL)	−0.560 **
Obliterated vessels (%)			
Small vessels	0.598 ***		
Medium vessels	0.420 *		
Large vessels	0.354		

* *p* < 0.05, ** *p* < 0.01 and *** *p* < 0.001. Spearman’s rank correlation test.

## References

[1] Hoeper M.M., Humbert M., Souza R., Idrees M., Kawut S.M., Sliwa-Hahnle K., Jing Z.C., Gibbs J.S. (2016). A global view of pulmonary hypertension. Lancet Respir. Med..

[2] Hoeper M.M., Humbert M. (2019). The new haemodynamic definition of pulmonary hypertension: Evidence prevails, finally!. Eur. Respir. J..

[3] Galie N., Humbert M., Vachiery J.L., Gibbs S., Lang I., Torbicki A., Simonneau G., Peacock A., Vonk Noordegraaf A., Beghetti M. (2016). 2015 ESC/ERS Guidelines for the diagnosis and treatment of pulmonary hypertension: The Joint Task Force for the Diagnosis and Treatment of Pulmonary Hypertension of the European Society of Cardiology (ESC) and the European Respiratory Society (ERS): Endorsed by: Association for European Paediatric and Congenital Cardiology (AEPC), International Society for Heart and Lung Transplantation (ISHLT). Eur. Heart J..

[4] Humbert M., Guignabert C., Bonnet S., Dorfmuller P., Klinger J.R., Nicolls M.R., Olschewski A.J., Pullamsetti S.S., Schermuly R.T., Stenmark K.R. (2019). Pathology and pathobiology of pulmonary hypertension: State of the art and research perspectives. Eur. Respir. J..

[5] Tuder R.M., Marecki J.C., Richter A., Fijalkowska I., Flores S. (2007). Pathology of pulmonary hypertension. Clin. Chest Med..

[6] Dotan Y., Stewart J., Gangemi A., Wang H., Aneja A., Chakraborty B., Dass C., Zhao H., Marchetti N., D’Alonzo G. (2020). Pulmonary vasculopathy in explanted lungs from patients with interstitial lung disease undergoing lung transplantation. BMJ Open Respir. Res..

[7] Waxman A., Restrepo-Jaramillo R., Thenappan T., Ravichandran A., Engel P., Bajwa A., Allen R., Feldman J., Argula R., Smith P. (2021). Inhaled Treprostinil in Pulmonary Hypertension Due to Interstitial Lung Disease. N. Engl. J. Med..

[8] Lee J., Song J.U. (2020). The Clinical Efficacy of Pulmonary Hypertension-Specific Agents in Idiopathic Pulmonary Fibrosis: Systematic Review and Meta-Analysis of Randomized Controlled Clinical Trials. J. Korean Med. Sci..

[9] Rajagopal K., Bryant A.J., Sahay S., Wareing N., Zhou Y., Pandit L.M., Karmouty-Quintana H. (2021). Idiopathic pulmonary fibrosis and pulmonary hypertension: Heracles meets the Hydra. Br. J. Pharmacol..

[10] Maron B.A., Leopold J.A. (2014). The role of the renin-angiotensin-aldosterone system in the pathobiology of pulmonary arterial hypertension (2013 Grover Conference series). Pulm. Circ..

[11] de Man F.S., Tu L., Handoko M.L., Rain S., Ruiter G., Francois C., Schalij I., Dorfmuller P., Simonneau G., Fadel E. (2012). Dysregulated renin-angiotensin-aldosterone system contributes to pulmonary arterial hypertension. Am. J. Respir. Crit. Care Med..

[12] Tsutsumi Y., Matsubara H., Ohkubo N., Mori Y., Nozawa Y., Murasawa S., Kijima K., Maruyama K., Masaki H., Moriguchi Y. (1998). Angiotensin II type 2 receptor is upregulated in human heart with interstitial fibrosis, and cardiac fibroblasts are the major cell type for its expression. Circ. Res..

[13] Wagenaar G.T., Laghmani El H., Fidder M., Sengers R.M., de Visser Y.P., de Vries L., Rink R., Roks A.J., Folkerts G., Walther F.J. (2013). Agonists of MAS oncogene and angiotensin II type 2 receptors attenuate cardiopulmonary disease in rats with neonatal hyperoxia-induced lung injury. Am. J. Physiol. Cell Mol. Physiol..

[14] Matavelli L.C., Siragy H.M. (2015). AT2 receptor activities and pathophysiological implications. J. Cardiovasc. Pharmacol..

[15] Sumners C., de Kloet A.D., Krause E.G., Unger T., Steckelings U.M. (2015). Angiotensin type 2 receptors: Blood pressure regulation and end organ damage. Curr. Opin. Pharmacol..

[16] Barber M.N., Sampey D.B., Widdop R.E. (1999). AT(2) receptor stimulation enhances antihypertensive effect of AT(1) receptor antagonist in hypertensive rats. Hypertension.

[17] Ferreira A.J., Shenoy V., Yamazato Y., Sriramula S., Francis J., Yuan L., Castellano R.K., Ostrov D.A., Oh S.P., Katovich M.J. (2009). Evidence for angiotensin-converting enzyme 2 as a therapeutic target for the prevention of pulmonary hypertension. Am. J. Respir. Crit. Care Med..

[18] Shenoy V., Kwon K.C., Rathinasabapathy A., Lin S., Jin G., Song C., Shil P., Nair A., Qi Y., Li Q. (2014). Oral delivery of Angiotensin-converting enzyme 2 and Angiotensin-(1-7) bioencapsulated in plant cells attenuates pulmonary hypertension. Hypertension.

[19] Breitling S., Krauszman A., Parihar R., Walther T., Friedberg M.K., Kuebler W.M. (2015). Dose-dependent, therapeutic potential of angiotensin-(1-7) for the treatment of pulmonary arterial hypertension. Pulm. Circ..

[20] Rathinasabapathy A., Bryant A.J., Suzuki T., Moore C., Shay S., Gladson S., West J.D., Carrier E.J. (2018). rhACE2 Therapy Modifies Bleomycin-Induced Pulmonary Hypertension via Rescue of Vascular Remodeling. Front. Physiol..

[21] Santos R.A., Simoes e Silva A.C., Maric C., Silva D.M., Machado R.P., de Buhr I., Heringer-Walther S., Pinheiro S.V., Lopes M.T., Bader M. (2003). Angiotensin-(1-7) is an endogenous ligand for the G protein-coupled receptor Mas. Proc. Natl. Acad. Sci. USA.

[22] Bosnyak S., Jones E.S., Christopoulos A., Aguilar M.I., Thomas W.G., Widdop R.E. (2011). Relative affinity of angiotensin peptides and novel ligands at AT1 and AT2 receptors. Clin. Sci..

[23] Steckelings U.M., Widdop R.E., Sturrock E.D., Lubbe L., Hussain T., Kaschina E., Unger T., Hallberg A., Carey R.M., Sumners C. (2022). The Angiotensin AT2 Receptor: From a Binding Site to a Novel Therapeutic Target. Pharmacol. Rev..

[24] Raud J. (2022). (VicorePharma AB, Stockholm, Sweden).

[25] Tornling G., Batta R., Porter J.C., Williams B., Bengtsson T., Parmar K., Kashiva R., Hallberg A., Cohrt A.K., Westergaard K. (2021). Seven days treatment with the angiotensin II type 2 receptor agonist C21 in hospitalized COVID-19 patients; a placebo-controlled randomised multi-centre double-blind phase 2 trial. EClinicalMedicine.

[26] VicorePharma Safety, Efficacy and Pharmacokinetics of C21 in Subjects With IPF. https://www.clinicaltrials.gov/ct2/show/NCT04533022.

[27] Bruce E., Shenoy V., Rathinasabapathy A., Espejo A., Horowitz A., Oswalt A., Francis J., Nair A., Unger T., Raizada M.K. (2015). Selective activation of angiotensin AT2 receptors attenuates progression of pulmonary hypertension and inhibits cardiopulmonary fibrosis. Br. J. Pharmacol..

[28] Rathinasabapathy A., Horowitz A., Horton K., Kumar A., Gladson S., Unger T., Martinez D., Bedse G., West J., Raizada M.K. (2018). The Selective Angiotensin II Type 2 Receptor Agonist, Compound 21, Attenuates the Progression of Lung Fibrosis and Pulmonary Hypertension in an Experimental Model of Bleomycin-Induced Lung Injury. Front. Physiol..

[29] Abe K., Toba M., Alzoubi A., Ito M., Fagan K.A., Cool C.D., Voelkel N.F., McMurtry I.F., Oka M. (2010). Formation of plexiform lesions in experimental severe pulmonary arterial hypertension. Circulation.

[30] Colvin K.L., Yeager M.E. (2014). Animal Models of Pulmonary Hypertension: Matching Disease Mechanisms to Etiology of the Human Disease. J. Pulm. Respir. Med..

[31] Derrett-Smith E.C., Dooley A., Gilbane A.J., Trinder S.L., Khan K., Baliga R., Holmes A.M., Hobbs A.J., Abraham D., Denton C.P. (2013). Endothelial injury in a transforming growth factor beta-dependent mouse model of scleroderma induces pulmonary arterial hypertension. Arthritis Rheum..

[32] Farkas L., Gauldie J., Voelkel N.F., Kolb M. (2011). Pulmonary hypertension and idiopathic pulmonary fibrosis: A tale of angiogenesis, apoptosis, and growth factors. Am. J. Respir. Cell Mol. Biol..

[33] Derrett-Smith E., Clark K.E.N., Shiwen X., Abraham D.J., Hoyles R.K., Lacombe O., Broqua P., Junien J.L., Konstantinova I., Ong V.H. (2021). The pan-PPAR agonist lanifibranor reduces development of lung fibrosis and attenuates cardiorespiratory manifestations in a transgenic mouse model of systemic sclerosis. Arthritis Res. Ther..

[34] Denton C.P., Spierings J. (2021). Combining Data Sets as Well as Therapies Shows Improved Outcome in Connective Tissue Disease-Associated Pulmonary Hypertension. Arthritis Rheumatol..

[35] Khanna D., Zhao C., Saggar R., Mathai S.C., Chung L., Coghlan J.G., Shah M., Hartney J., McLaughlin V. (2021). Long-Term Outcomes in Patients With Connective Tissue Disease-Associated Pulmonary Arterial Hypertension in the Modern Treatment Era: Meta-Analyses of Randomized, Controlled Trials and Observational Registries. Arthritis Rheumatol..

[36] Blagojevic J., Abignano G., Avouac J., Cometi L., Frerix M., Bellando-Randone S., Guiducci S., Bruni C., Huscher D., Jaeger V.K. (2020). Use of vasoactive/vasodilating drugs for systemic sclerosis (SSc)-related digital ulcers (DUs) in expert tertiary centres: Results from the analysis of the observational real-life DeSScipher study. Clin. Rheumatol..

[37] Jaeger V.K., Wirz E.G., Allanore Y., Rossbach P., Riemekasten G., Hachulla E., Distler O., Airo P., Carreira P.E., Balbir Gurman A. (2016). Incidences and Risk Factors of Organ Manifestations in the Early Course of Systemic Sclerosis: A Longitudinal EUSTAR Study. PLoS ONE.

[38] Puchtler H., Waldrop F.S., Valentine L.S. (1973). Polarization microscopic studies of connective tissue stained with picro-sirius red FBA. Beitr. Pathol..

[39] Gilhodes J.C., Jule Y., Kreuz S., Stierstorfer B., Stiller D., Wollin L. (2017). Quantification of Pulmonary Fibrosis in a Bleomycin Mouse Model Using Automated Histological Image Analysis. PLoS ONE.

[40] Testa L.C., Jule Y., Lundh L., Bertotti K., Merideth M.A., O’Brien K.J., Nathan S.D., Venuto D.C., El-Chemaly S., Malicdan M.C.V. (2021). Automated Digital Quantification of Pulmonary Fibrosis in Human Histopathology Specimens. Front. Med..

